# Cost and Effort Considerations for the Development of Intervention Studies Using Mobile Health Platforms: Pragmatic Case Study

**DOI:** 10.2196/29988

**Published:** 2022-03-31

**Authors:** Dan Thorpe, John Fouyaxis, Jessica M Lipschitz, Amy Nielson, Wenhao Li, Susan A Murphy, Niranjan Bidargaddi

**Affiliations:** 1 Flinders Digital Health Research Lab College of Medicine and Public Health Flinders University Clovelly Park Australia; 2 Brigham and Women's Hospital Harvard Boston, MA United States; 3 Radcliffe Institute Harvard University Boston, MA United States

**Keywords:** health informatics, human computer interaction, digital health, mobile health, ecological momentary assessment, ecological momentary intervention, behavioral activation, interventional research, mobile health costs

## Abstract

**Background:**

The research marketplace has seen a flood of open-source or commercial mobile health (mHealth) platforms that can collect and use user data in real time. However, there is a lack of practical literature on how these platforms are developed, integrated into study designs, and adopted, including important information around cost and effort considerations.

**Objective:**

We intend to build critical literacy in the clinician-researcher readership into the cost, effort, and processes involved in developing and operationalizing an mHealth platform, focusing on Intui, an mHealth platform that we developed.

**Methods:**

We describe the development of the Intui mHealth platform and general principles of its operationalization across sites.

**Results:**

We provide a worked example in the form of a case study. Intui was operationalized in the design of a behavioral activation intervention in collaboration with a mental health service provider. We describe the design specifications of the study site, the developed software, and the cost and effort required to build the final product.

**Conclusions:**

Study designs, researcher needs, and technical considerations can impact effort and costs associated with the use of mHealth platforms. Greater transparency from platform developers about the impact of these factors on practical considerations relevant to end users such as clinician-researchers is crucial to increasing critical literacy around mHealth, thereby aiding in the widespread use of these potentially beneficial technologies and building clinician confidence in these tools.

## Introduction

Interest in integrating mobile technology into research has grown exponentially over the last 2 decades, particularly as a means of collecting real-time observational data and intervention outcomes [1]. A newer, but equally growing area of research involves Just-In-Time Adaptive Interventions (JITAIs) [2], which use data collected via mobile/wearable devices, such as symptoms and behaviors, to identify opportune moments for delivering personalized interventional content in real time.

These increasingly popular techniques have created a thriving marketplace for mobile health (mHealth) platforms designed to aggregate mobile app data, deliver intervention content, and observe outcomes. For example, the open-source AWARE framework is an Android platform designed to address the lack of availability of open and reusable software for creating context-aware apps on mobile devices [3]. Similarly, Remote Assessment of Disease and Relapse-base is a scalable, fully functional, remote Internet of Things data collection platform for real-time remote sensor data collection [4]. Such platforms allow clinicians and researchers to collect (1) ecological momentary assessment (EMA) data, for example, active self-report surveys collected on a mobile or wearable device; (2) sensor data from external devices, such as data collected by a wrist-worn device like a Fitbit [5] or a mattress sensor like the Withings Sleep Analyzer [6]; and (3) sensor data directly collected from participants’ smartphones, including step counts, geolocation data throughout the day, and so on.

Clinician-researchers seeking to integrate mobile app data into their research or practice either to deliver interventions or to monitor existing interventions have a plethora of options. However, very few apps include all these features, and implementing these apps on a large scale can be problematic without specific technical and implementation knowledge [4,7]. Should one wish to learn these skills, there is limited documentation with respect to understanding the pragmatic processes required for setting up these studies, building new features, and assessing the feasibility of technical solutions to these research questions. Furthermore, for many clinician-researchers, this is a decision that is outside of their realm of expertise. A wrong decision can dramatically impact the feasibility of meeting a given set of research aims. Indeed, despite the availability of several mHealth platforms, guidance for integrating intervention aims with technical capabilities—from a combined technical and implementation perspective—is difficult to find and would be valuable to researchers working with these tools.

To contribute to this body of knowledge, this paper describes the programmatic processes involved using our experiences with developing an mHealth platform, Intui. The mHealth platform is described with a particular focus on the decisions and choices of relevance to health services and clinician-researchers, including the procedure, cost and effort, and design choices at the product and project levels. To illustrate this further, we present the software results of these processes in action, namely a practical example of the adaptation of Intui to a behavioral activation therapy context, adding practical depth and detail to our procedures.

## Methods

### Materials

#### Intui Platform: Design Decisions and Features

**Figure 1:**
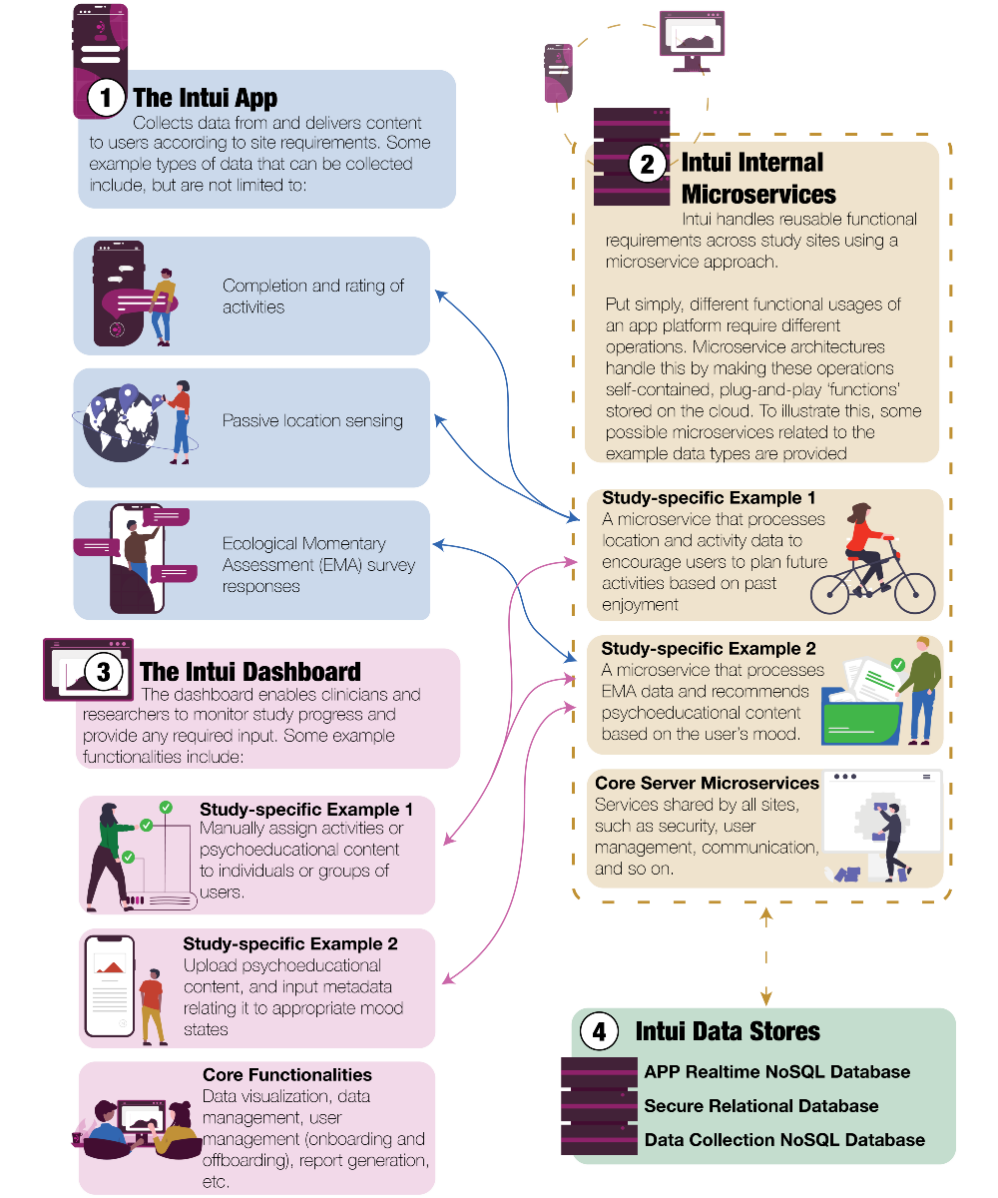
Components, architecture, and example functionalities of the Intui platform.

The Intui platform is a configurable cloud-based service designed to support mHealth data collection and intervention studies (Figure 1). The 4 core software elements of the platform are (1) the Android and iOS smartphone apps for participant use; (2) lightweight cloud functions, also known as microservices, to handle reusable functional requirements provided in the app and the dashboard—for example, security, user management, communication, data management and data analysis—in isolation and at scale; (3) the web-based data dashboard for clinician-researchers to manage study participants and data; and (4) cloud storage on Amazon or Google data centers.

The app and dashboard user interfaces are implemented in a cross-platform app development framework, Ionic, which allows us to create and implement modular components that can be reused in multiple projects as required. Ionic also supports easy integration of third-party plug-ins, which can enable rapid development of new features [8]. For back-end functionality and data storage, Google and Amazon cloud services are used owing to their easy scalability and ability to integrate functionalities implemented in a number of programming languages as microservices.

### Study Design and Initiation

The process of initiating a new study within the Intui platform consists of three parts, guided by the Intui platform software development team: (1) setting up data hosting, (2) configuring Intui to the study-specific requirements, and (3) developing and implementing any custom study components.

#### Data Hosting Setup

The data hosting setup begins with an Intui software developer preparing the study-specific databases. The Intui platform is designed such that each study has a separate set of databases, 1 set for storing deidentified study data and another for securely storing personally identifiable participant details in an encrypted format. This provides an assurance that data segregation exists from 1 study to another for privacy and ethical considerations, as discussed in detail later. At this stage, a unique study code is also generated for registered clinician-researchers.

#### Study-Specific Configuration

Once the hosting setup is complete, registered clinician-researchers receive a welcome email with details to access the web-based data dashboard, as well as the study code to share with prospective participants using the app. Researchers can also use the data dashboard to perform administrative functions, such as inviting participants, removing participants, downloading data, viewing summary statistics, and monitoring study progress.

Based on the unique requirements of each study, Intui software developers extract the study-specific configurations. These configurations define the inclusion of existing functionalities and components of the Intui platform as well as any parameters required for them to operate within the research design. For low-complexity studies, this includes the data collection requirements for phone sensor data and external device sensors, the definition of EMA question sets with branching logic, and protocol details such as the length of the study and timing of reminders related to the proposed data collection.

#### Development and Implementation of Any Custom Study Components

High-complexity studies often involve the implementation of custom study components. These components may include app user interface (UI) elements for intervention delivery, custom-built data gathering functionalities, and the implementation of microservices to handle the functional logic requirements of more complex study designs. Intui software developers work with researchers to define, design, and implement these components, or integrate those developed by third parties. This flexibility allows researchers to retain full software development control over aspects of more complex study designs.

In addition, UI components that encompass visual elements (styling and display), data (what is shown), and logic (what needs to be stored and any conditional functionality) may need to be developed. These can be designed by the Intui platform developers or by an external developer. Once a prototype UI component is available, the Intui platform developers integrate the component into the Intui app and release an updated version of the app with all added functionalities.

When a study has completed the initiation process, Intui software developers collaborate with the study researchers to run a short technical pilot. The pilot period validates the functional components and configured settings for the study ahead of participant recruitment. Technical pilots ensure that the right data are collected and that the study design has been correctly implemented to reduce the risk of participant dropout as a result of technical faults.

### Measures

#### EMA Delivery

The design of the Intui mHealth platform permits the administration of various EMA question types and tailoring of the question flow and timing. For each study, the EMA question set(s) are defined within a study-specific configuration, and the schedule for question delivery is set up within the study protocol microservice of the Intui platform. EMA question types can include numerical sliders, Likert scales, single choice (eg, yes/no), multiple choice, and free-text input. Branching logic can be used to set conditions on which questions are shown. For example, if poor sleep is self-reported in the app, an additional question may appear asking for further information to differentiate difficulties in falling asleep.

#### Sensor Measures

The sensor data types and sources collectible through Intui are, essentially, unlimited. Sensors can be integrated with Intui at the application programming interface (API) level and through manual data entry methodologies, for example, participants entering results from an analog medical device.

### Procedure

#### Participant Onboarding

Study participants are invited to download the Intui app from the respective Apple-iOS or Google-Android app stores. Participants register on the Intui app using the provided study code. The unique study code enables the study-specific configuration and features within the app and may also be used to link or segregate participant data. For example, a study may require the use of a patient app that feeds data into a clinician dashboard. In this scenario, clinicians are assigned a unique study code that they provide to participants, thereby linking multiple patient apps to an individual clinician’s dashboard. This ensures that only authorized clinicians can access patient information linked to their study code, maintaining data segregation.

### Collection of EMA Data

The timing of administering EMAs may be based on time-contingent, event-contingent, or answer-contingent schedules. Time-contingent schedules are configured at a predetermined time of the day/week (for example, a survey getting sent at 2 PM every Monday) or within a range of possible times (for example, a prompt being set to be sent at some point between 9 AM and 3 PM). Event-contingent schedules deliver a tailored prompt in response to characteristics, trends, or threshold values identified in the collected data (when event X occurs, nudge Y). Event-contingent nudges can be delivered in response to active data (when a self-reported threshold value is recorded or a trend is observed over several days) or passive data (like arriving at a specific location, reported through a smartphone’s GPS sensor). The type of questions posed in the app and the schedule for delivering those questions can be customized to the requirements of each specific study. Additionally, the appearance of questions may be conditionally based on responses to previous questions (eg, if response is A, ask B). For example, if poor sleep is self-reported in the app, an additional question may appear asking for further information to differentiate difficulties in falling asleep.

The EMA question set(s) are defined within study-specific configuration settings and the schedule for question delivery is set up within the study protocol microservice of the Intui platform for each study.

#### Delivery of JITAIs

Access to data in real time is critical to the implementation of JITAIs, as is the development of the necessary UI components to enable this implementation. External and internal programmers can access data gathered in real time through the secure data RESTful APIs in Intui. This can facilitate rapid and collaborative intervention development, as programmers can prototype approaches to process data in any environment (Python, R-Studio, Java, JavaScript) and schedule algorithms to run as independent applications (microservices) that use the same Intui database to read data and store processed results. Programmers can also use the Intui API, which can control push notifications to users and set up reminders to access interventions within the app. Such interventions can include brief text within push notifications, multimedia content within the app, or a set of EMA questions.

#### Data Extraction for Ongoing Analysis

The collected participant data may be downloaded at any time from the clinician-researcher dashboard or using the Intui API. Clinician-researchers can download data in the CSV format for offline analysis at any time from the data dashboard. Alternatively, separate data analytic applications can be implemented to programmatically read and process the gathered study data through APIs in real time and initiate study features (eg, present new content or messages to participants).

### Considerations Unrelated to the Study Design

#### Support

Researchers can expect to receive stable support for the duration of their study from the Intui platform development team. Owing to the monthly support and maintenance fee, researchers (and study participants) can submit bug reports and expect support and resolution within reasonable time frames to ensure the continuous and uninterrupted progress of their study. The Intui platform development team includes app and cloud computing software developers who hold grant-funded project positions and are also working in the industry. These developers work with the lab on 1 or more projects in an ongoing manner.

#### Privacy and Ethics

Researchers need to establish an agreement with Flinders University to use the Intui platform that will outline the terms of hosting and sharing of data between the Intui development team and researchers. All data collected by the Intui app are transferred to and stored in 2 databases to lower the impact of a potential data security breach “at rest.” Personal identifiable data (eg, names, email addresses used for registration, and logins) are stored in an encrypted user management database in Amazon Web Services data centers. Data collected and used in the study such as questionnaire/survey data and passive data streams are stored on Google Cloud Firestore. Due to the possibility of identifying individuals through their location data coordinates, all raw location data are encrypted before leaving the phone and remain encrypted during storage within the database in a format not readable by humans.

#### Cost and Effort

In the interest of transparency, we have provided the following example data in Australian Dollars (AUDs) associated with previous projects. The following conversion rate was used: AUD $1=US $0.72. In short, setup costs depend on the functionalities required by the study, the associated software development effort required for the app, and for any back-end development required on the functional database. This is further influenced by the complexity of the project, its duration, and the number of participants.

 A low-complexity study that makes use of existing functionalities and components incurs the standard Intui platform study setup effort of 12 hours, roughly costed at AUD $100 per hour for the example in this paper, leading to a total of AUD $1200. This includes advice on study design and consultation with the team to determine study requirements. The baseline costs associated with technical support and maintenance efforts are 3 hours per month (approximately AUD $300) for studies involving up to 2000 participants.

In addition to these labor hours, infrastructure costs—like data hosting and cloud services—start from an AUD $100 per month allocation for studies of up to 2000 participants. Infrastructure costs for serverless environments are often difficult to estimate a priori* *but are based here on the Google Cloud Platform hosting costs at the time of printing (2021). Free credit tiers may influence infrastructure costs, for example, Cloud Function free tier of 2 million invocations per month, and Firebase free tier of 50,000 reads and 20,000 writes per day. The baseline allocation set here is used to cover Google Cloud resources used by App Engine for storage, logging, and reading operations, or where Cloud Function and Firebase usage exceeds free tier levels on heavy use days; however, this should be monitored closely during the early stages of any project to adjust for redundant usage and identify infrastructure cost savings at scale.

For a practical example involving a simple study, Intui was used to collect third-party device data over a 6-month observational study investigating the relationship between mental health and device data of at-risk young adults for a total cost of AUD $3000 [7]. In this study, participants installed the Intui app on their phone that extracted daily, passively recorded active time, and administered EMAs addressing participants’ mood, sleep, and eating habits.

Higher complexity studies are costed on a time and effort basis. In this context, complexity can include the development and integration of custom UI elements, developing and ensuring high-quality integration of microservices that can handle custom intervention logics, or any other request that requires substantial effort beyond the hours outlined in the more basic worked example. The case study presented later in this paper provides a good worked example of the costs associated with a more complex project. Additional costs may also be incurred for alterations to the study design and data collection while a study is running, technically referred to as change requests. Studies with over 2000 participants incur additional monthly costs to account for more extensive support, maintenance, and infrastructure requirements.

## Results

### App Implementation Using Intui

To make these considerations less abstract, we illustrate how the Intui platform was used to implement an app to support a behavioral activation (BA) intervention. BA is an evidence-based technique used predominantly to manage depressive symptoms. The aim of BA is to increase engagement in behaviors that promote feelings of pleasure and mastery [9]. This technique requires participants to keep an accurate record of activities undertaken over a period and report their mood while undertaking that activity. The goal of this technique is for participants to learn to identify and schedule enjoyable and mastery-oriented activities. However, compliance is a recognized barrier to this treatment method, and few participants complete the prescribed protocol [10].

**Figure 2:**
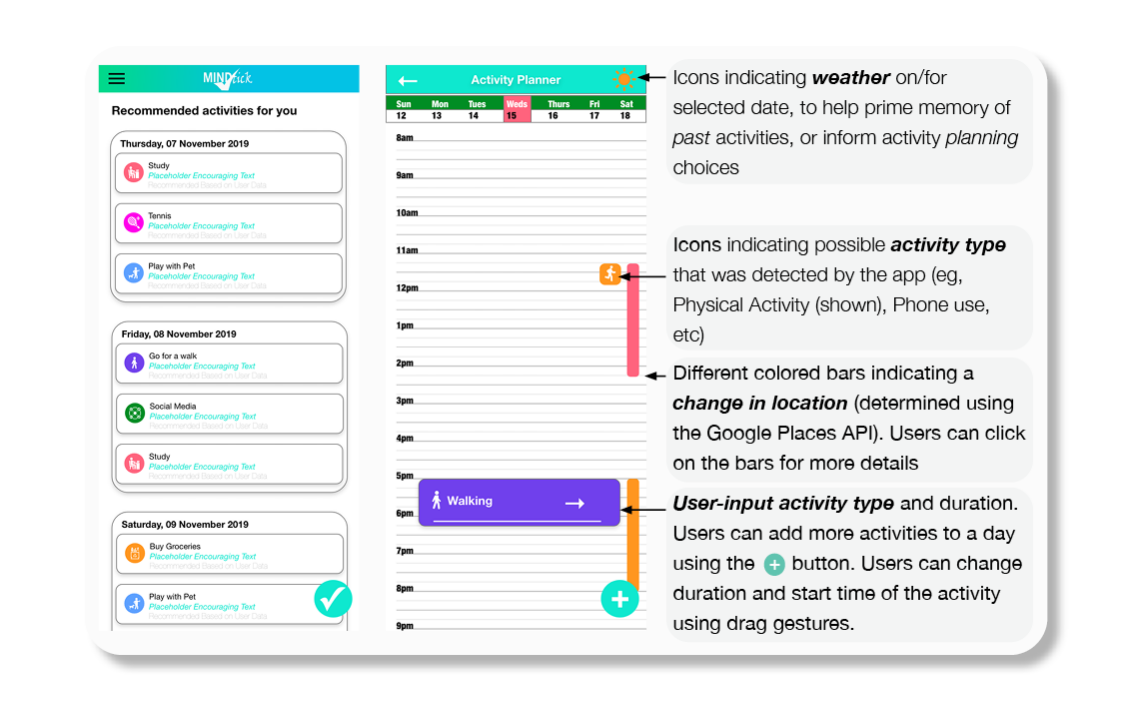
Intui behavioral activation implementation, including an activity recommendation (left) and a planning/logging (right) interface.

We implemented a BA app within the Intui platform with adaptive interventions to reduce the burden of monitoring and planning (Figure 2). This was achieved through adaptation of existing components and through the development of two new interventional components: (1) a nudging intervention to improve recall, planning, and adherence for self-monitoring; and (2) machine learning–assisted activity planning.

### Study Design and Initiation

#### Data Hosting Setup

We configured a study-specific database on Google Firestore. User-reported data are stored in and recommended activities are fetched from the same study database. Additionally, we configured the app to collect location coordinates every few minutes and store them in the study database. We used a secure data API to read from and write to the study database. Next, we implemented 2 internet-based algorithms (described below) to read from and write to the study database to operationalize the 2 real-time intervention components.

#### Study-Specific Configuration

Pre-existing EMA components within Intui, as well as much of the back end and UI components of the app and dashboard, were reused or adapted for this project.

#### Development and Implementation of Any Custom Study Components

The BA app offered 2 interventional components using real-time data, namely nudging to improve recall and adherence to self-monitoring, and machine learning–assisted activity planning. These interventional components were designed specifically for this study, and they were crucial to its success. Outside of the algorithm development, the implementation and development of the custom app UI calendar elements, app UI location context detail elements, and the implementation of microservices to handle the logic for the recommender algorithm as well as the location clustering algorithm also contributed substantially to the development effort.

### Interventions Developed

The first intervention component aims to enhance the user’s ability to retrieve previously experienced activities and moods as part of self-monitoring. It achieves this by nudging relevant spatial and behavioral cues, involving push notifications and in-app cues to predictably alter user behavior in a way that is predictable but without specifically forbidding any options or producing disincentivizing effects [11]. Using a calendar or list view interface in the app, users keep a detailed log of their past activities and associated moods. When a user clicks in the calendar to make an activity entry for any given day and time, contextual cues mined from the mobile phone location and usage data are displayed. The cues, shown as visual elements (Figure 2), correspond to where the users were (derived from location data, as described below), what activities they were doing (derived from movement sensor data), and the environment (weather of the location); all these are possible, thus aiding more accurate recall [12,13].

**Figure 3:**
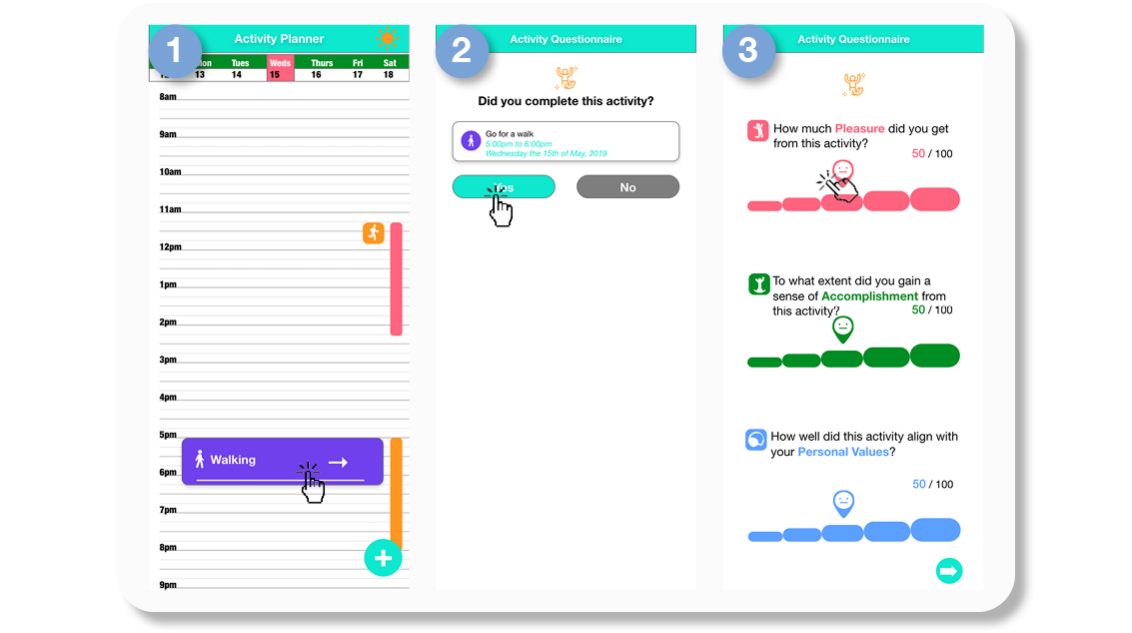
Intui behavioral activation app activity evaluation questionnaire. After inputting an activity or clicking on a machine learning–generated activity suggestion in the planner view (Step 1), users are asked to confirm the details of the activity (Step 2), and then answer 3 short questions evaluating the activity (Step 3). API: application programming interface.

For cueing users to recall their activities and mood more accurately when they were recording data, we displayed visited places as vertical bars in the daily calendar view. Each bar visualized the start and end times of 1 visited place (Figures 2-3). When the bar was clicked, the users were able to see a map and the details of each location, including the precise start and end times. These vertical bars were generated daily, using the results of a k-means clustering algorithm that was set to run at midnight. This algorithm works by taking a continuous stream of location coordinates gathered from the phone sensors, clustering them into locations, and then outputting them as a list of visited places. Entries for the start and end times of each location, and the central GPS point of the cluster marked as the visited place were made into the database. Once users confirm the type of activity they have completed, they are asked to evaluate the activity through a short questionnaire (Figure 3).

The second intervention component aims to assist with planning activities by making personalized activity recommendations learned from the users’ self-monitored data and that of other users with similar behavioral patterns. The planner interface displays a ranked order list of activity recommendations drawn from a mix of previously recorded activities and random activities (Figure 2). Users select an activity and schedule when to undertake it in the future. Users’ responses to the suggested activities and their evaluation are processed through a bilinear model to refine the ranked order listing of future recommendations [14]. Over time, the recommender interface personalizes the ranked order list with higher-order ranking for activities that reflect long-term user preferences; that is, activities that have resulted in better mood outcomes are the most likely ones to be completed.

To ensure that activity suggestions were personalized to user circumstances, the order of activities displayed in the planner view were dynamically ranked based on past data (Figure 3). To achieve this, we implemented the linear upper confidence bound algorithm, which weighs activities reaping the most rewards (in terms of mood and likelihood to be completed) when exploring new options that may be equally or more rewarding; this is a very important consideration in the context of BA, where targeting reward responsiveness is crucial [15]. The algorithm takes user activity preferences from past data entries and a batch model trained using the first 8 weeks of data from all the users, and it outputs a ranked list of activities to help users prioritize activities that may reward them. A second microservice was scheduled to refresh the batch model daily at midnight after an 8-week period.

### Cost and Effort

These algorithms were implemented in Python and run as independent microservices on an Ubuntu instance. This work was carried out independently by a research assistant with a background in data science and Python programming. The specifications for storing the results of the algorithms were established by the platform developers. Additionally, these platform developers designed and implemented the UI elements that incorporated the outputs of these algorithms into the clinician-researcher dashboard. This case study was completed in 62 hours (costed at AUD $100 per hour for $6200).

## Discussion

### Principal Findings

The clinician-researchers seeking to integrate mobile app data into their research either to deliver interventions or to monitor existing interventions have a plethora of options. As evidenced by the overall process of developing Intui and the case study outlined in this study, the considerations clinician-researchers must bear in mind when setting out to perform this research are extensive. However, Intui represents just 1 approach to designing, developing, and implementing these tools, and regardless of the platform, there is a need to reflect on the ability of clinician-researchers and their teams to meet the logistical and technical requirements underpinning the success of these platforms in their contexts.

Ecological studies, especially longitudinal studies, often require extensive infrastructure and information technology support that demand careful planning to ensure high-quality development, implementation, and maintenance [16]. The frequency at which extremely granular data are stored, received, segmented, analyzed, and presented is also a challenge that can be underestimated, thus affecting the ability of platforms to be used for multiple research outcomes. All these decisions drastically impact the feasibility of delivering projects, are difficult to plan for and cost without a nontechnical background, and can either limit or enable the possibilities of a project. Indeed, this is critical to the provision of the support we have integrated into our operations as nonnegotiable for Intui.

These issues are not confined to our experiences in developing Intui and supporting trials using it; indeed, cost and effort—including the up-front and opportunity costs due to the perceived loss of time in learning new tools—training, and support, were listed as prescient concerns by de Grood and colleagues in their scoping review of eHealth technology adoption by physicians [17]. Open-sourcing projects claims to address these concerns in many respects, namely through literal transparency by sharing the bare bones of software for all to scrutinize [18]. In an age of WebMD self-diagnoses, we are assured that our medical audience requires little convincing that open-sourcing data only results in transparency of the pragmatic type if it is received by an audience able to understand, contextualize, and critique it [7,19,20]. In the same sense that health consumers are ultimately justified in their desire to combat medical gatekeeping and the harm it can cause [7,19], health professionals have every right to question the authority and hegemony of software developers and technology companies on eHealth, especially given the questionable quality of these software packages sometimes [21-23]. However, the solution in both cases lies on a middle ground; solutions can be developed through new models with transparent collaboration and through the development of a critical audience capable of engaging with these digital realities in a reflexive and an ongoing manner [7,20,24]. Through providing insights into the cost, effort, and processes involved in developing and operationalizing Intui in this paper, we hope to have contributed to the development of this critical audience as well as to more transparent norms in mHealth platform development and operationalization in a broader sense.

### Conclusions

Enabling researchers to rapidly implement mobile data collection and intervention studies can ultimately lead to increased understanding and access to evidence-based behavioral and mental health interventions. Based on a case study involving our own platform and on critical reflection, this paper has outlined some practical considerations for those hoping to engage in these technologies. Greater transparency in terms of the cost, effort, and support across the board in this field will only enable the widespread use of these beneficial methodologies and build clinician confidence in these tools.

## Abbreviations

API: application programming interface
BA: behavioral activation
EMA: ecological momentary assessment
JITAIs: Just-In-Time Adaptive Interventions
mHealth: mobile health
UI: user interface
